# Electronic interaction in composites of a conjugated polymer and carbon nanotubes: first-principles calculation and photophysical approaches

**DOI:** 10.3762/bjnano.6.115

**Published:** 2015-05-08

**Authors:** Florian Massuyeau, Jany Wéry, Jean-Luc Duvail, Serge Lefrant, Abu Yaya, Chris Ewels, Eric Faulques

**Affiliations:** 1 Institut des Matériaux Jean Rouxel, Université de Nantes, CNRS, UMR6502, 2 rue de la Houssinière, 44322 Nantes, France; 2 Department of Materials Science and Engineering, University of Ghana, P.O. Box 24, Legon, Accra, Ghana

**Keywords:** composite, conjugated polymer, DFT calculations, energy transfer, photoconductivity, single wall carbon nanotubes, time-resolved photoluminescence

## Abstract

The mechanisms that control the photophysics of composite films made of a semiconducting conjugated polymer (poly(paraphenylene vinylene), PPV) mixed with single-walled carbon nanotubes (SWNT) up to a concentration of 64 wt % are determined by using photoexcitation techniques and density functional theory. Charge separation is confirmed experimentally by rapid quenching of PPV photoluminescence and changes in photocurrent starting at relatively low concentrations of SWNT. Calculations predict strong electronic interaction between the polymer and the SWNT network when nanotubes are semiconducting.

## Introduction

Electroactive conjugated polymers (ECPs) are technologically promising for organic light emitting diodes and photovoltaic cells [1–2]. Tuning the optical and conductive properties of ECPs is possible either by tailoring the polymer backbone with chemical side groups [3] or by preparing composites with nanoparticles [4–7]. ECP hybrids containing carbon nanotubes, fullerene-based molecules, and semiconducting nanocrystals have been extensively studied, in order to understand the photophysical changes observed on varying the particles concentration [8–11]. Notably, polymer-composite materials with different electron affinity have been used to enhance charge separation upon photoexcitation. The efficiency of this approach is strongly dependent on the internal junctions between the polymer and the electron acceptor or donor. However, many fundamental questions remain regarding the underlying energy transfer processes involved. Phase separation within domains should not in principle exceed the exciton diffusion length [12], which is the case for ECP–fullerene-based solar cells in which fullerene molecules are able to capture the negative charges while the holes remain on the conjugated polymer [13]. Still, evidence for similar photoinduced charge-transfer or energy-transfer mechanisms between conjugated polymers and carbon nanotubes blended at various concentrations is not yet established [14]. Poly(paraphenylene vinylene) (PPV) constitutes an excellent model system for ab initio calculations and photophysical experiments since it exhibits both highly efficient emission in the green-yellow range, and photoconduction under UV or blue light excitation.

In this report we present an investigation of the photoexcitation and exciton migration processes in composites of PPV and single-walled carbon nanotubes (SWNT) by means of time-resolved photoluminescence (PL) and photoconductivity measurements. These techniques are appropriate tools to understand the energy transfer mechanisms involved by the introduction of SWNTs into the PPV polymer matrix. In particular, time-resolved PL measurements give crucial information about the nature of photogenerated charges and their migration across the composite material. Our SWNTs batches are not sorted and contain both metallic and semiconducting (SC) nanotubes, as usual in most of the studies reporting about SWNT/polymer composites. These two kinds of SWNT species exhibit different electronic properties since SC nanotubes present a bandgap depending of their chirality. This fundamental physical difference will have tremendous importance as concerns the electronic coupling, the energy transfer and the migration of excitons between the semi-conducting polymer and the nanotubes [15]. Therefore, the results are compared with original density functional theory (DFT) calculations of coupling effects between the polymer and both species of SWNTs. Combined experimental results and first-principles calculations provide evidence that significant electronic interaction can take place between PPV chains and semiconducting SWNTs while metallic nanotubes are not coupled. This fundamental observation has strong relevance for applications.

## Results and Discussion

SWNT were purchased from Sigma Aldrich with a nominal purity of 50–70% and mean diameters 1.2–1.5 nm. The polymer precursor, poly(*p*-xylene tetrahydrothiophenium chloride), was mixed with SWNT in methanol as solvent. PPV–SWNT composite films of 200 nm thickness were obtained with SWNT mass concentrations *x* = 0–64% by drop casting onto ultraclean quartz substrates followed by thermal conversion under vacuum at 573 K.

Time-resolved PL experiments were carried out by exciting the samples at 4.64 eV with a 100 fs pulsed laser at low photon density (below 10^17^ cm^−3^) and PL images were recorded with a streak camera [16–17]. Steady-state PL spectra and quantum yields *Q* were measured at 3.1 eV excitation energy [18]. Photoconductivity measurements were performed at 2.54 eV with a high sensitivity Keithley electrometer.

**Figure 1:**
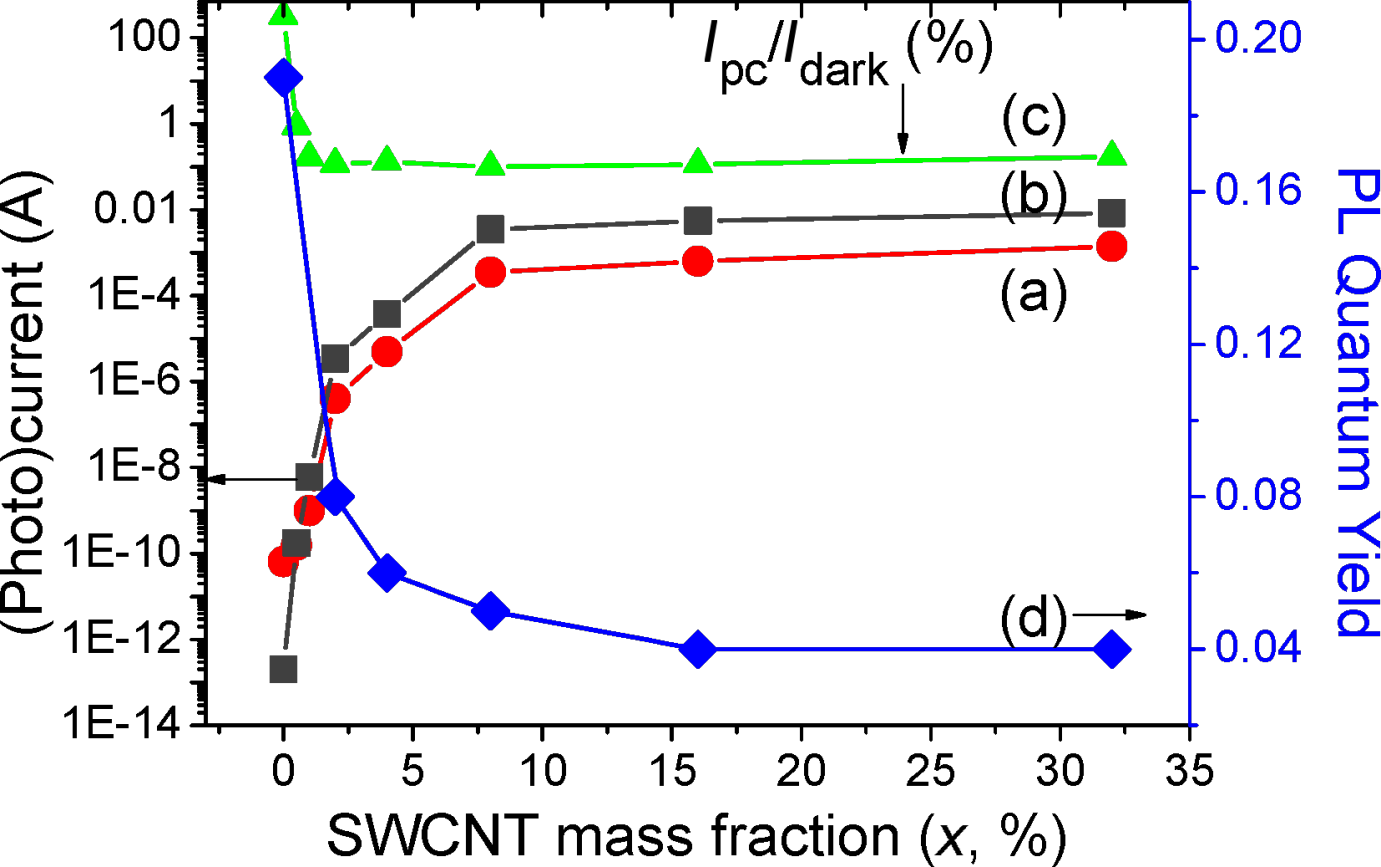
PL quantum yields and photoconductivity data of PPV/SWNT composite films converted at *T*_c_ = 573 K. (a) photocurrent *I*_pc_ = *I*_measured_− *I*_dark_; (b) dark current *I*_dark_; (c) ratio *I*_pc_/*I*_dark_; (d) PL quantum yields.

Figure 1 compares photoconductivity (PC) data and quantum yield estimates for the composite PPV/SWNT series *x* = 0–32%. We observe that the quantum yield decreases from a value of 19% (*x* = 0, standard PPV) in good agreement with the literature [19–20] down to 4% for *x* = 32% (Figure 1d). The photoconductivity measurements show a percolation regime beginning at a low threshold of *x* = 2% (see Figure 1a). A drastic quenching of PL occurs simultaneously with a substantial rise of the dark current and photocurrent spanning several orders of magnitude.

We suggest that the observed photoconductivity and PL behavior can be attributed to the dissociation of excitons and migration of charges onto the SWNTs. Other composite nanomaterials such as MEH-PPV/CdS, CdSe, MEH-PPV/silica, and PPV/TiO_2_ show a similar PL quenching when the mass percentage of nanomaterials introduced into the conjugated polymer matrix increases [4–6].

Figure 1a and Figure 1b show that the variations of dark current *I*_dark_ (without illumination) and photocurrent *I*_pc_ against SWNT concentration in the composite are exactly matched (*I*_pc_ is the difference between the measured photocurrent under illumination and the dark current). Since it is implicitly assumed that *I*_dark_ arises from the conductivity of quasi-metallic SWNTs, the change of *I*_pc_ vs *x* can be ascribed to the metallic character of the tubes. The assumption is that the semiconducting tubes are not active in dark current transfer, since photoexcitation would be required to produce mobile charge carriers in the tubes. This assumption is valid assuming that the Fermi level of the semiconducting tubes remains within their band gap, i.e., that there is no charge-transfer doping from the surrounding environment. Through density functional calculations we explore below this assumption, and show that it is not valid; the semiconducting tubes are in fact rendered semi-metallic due to interaction with nearby PPV.

Figure 1c shows that the ratio *I*_pc_/*I*_dark_ drops dramatically at very low nanotube concentrations, typically between 1 and 2% indicating that the dark current dominates the photocurrent above this threshold with conduction paths provided solely by highly conductive carbon nanotubes. In consequence PC data demonstrates an increasing density of charge carriers on SWNTs when *x* increases.

In order to understand the nature of PPV–nanotube interactions further we performed density functional (DFT) calculations under the local density approximation as implemented in the AIMPRO code [21–22], on a triphenyl PPV section (C_22_H_18_) oriented parallel to the axis of a metallic (4,4) and semiconducting (7,0) nanotube chosen due to their similar diameters. PPV is arranged axially along the SWNT following a previous study, in which combined angular-dependent Raman spectroscopy and DFT calculations demonstrated this is the energetically preferable orientation [23]. A hexagonal supercell was used containing a C_160_ (24.41 Å) section of (4,4) or a C_168_ (25.35 Å) section of (7,0) nanotube. The cell size was chosen so that nanotube axis were 27 Å apart to avoid interaction between neighboring tubes, and while the tube was held fixed the PPV structure was geometrically optimized. Calculations used a single k-point and Gaussian basis sets with 38 independent functions per carbon with Hartwigsen, Goedecker and Hütter relativistic pseudopotentials [24]. A Fermi temperature smearing for the electron population of *kT* = 0.001 eV was used. While the tubes modelled here have smaller diameters than those used in the experiment (for computational efficiency), we note that the reduced bandgap due to LDA to some extent counteracts what would otherwise be an overestimation in the modelling of the band gap of the experimental tubes. Specifically, we note that our calculated bandgap for the (7,0) nanotube of 0.08 eV is considerably underestimated compared to experiments, due to the well-known limitations of the LDA approximation. It is nonetheless in agreement with previous LDA calculations [25–27], notably with studies [27] showing that full structural and lattice optimization drops the calculated LDA band gap from around 0.5 eV [27–28] to around 0.2 eV.

**Figure 2:**
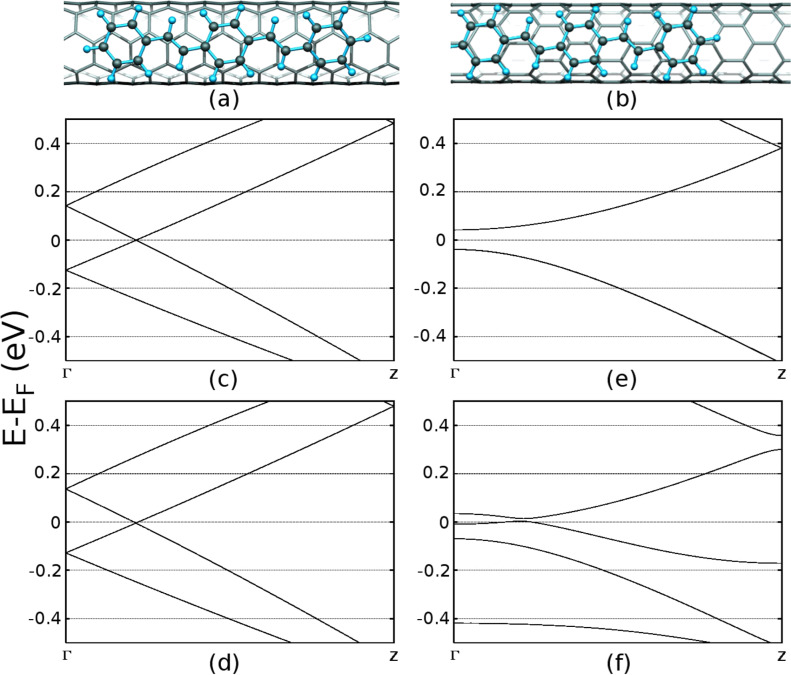
DFT calculations of triphenyl PPV-nanotube interaction. (a) metallic (4,4) nanotube, (b) semiconducting (7,0) nanotube. (c–f) show band structures in eV for (c,e) the isolated (4,4) and (7,0) nanotube sections, and (d,f) in conjunction with the PPV section. Fermi levels are aligned at zero.

**Figure 3:**
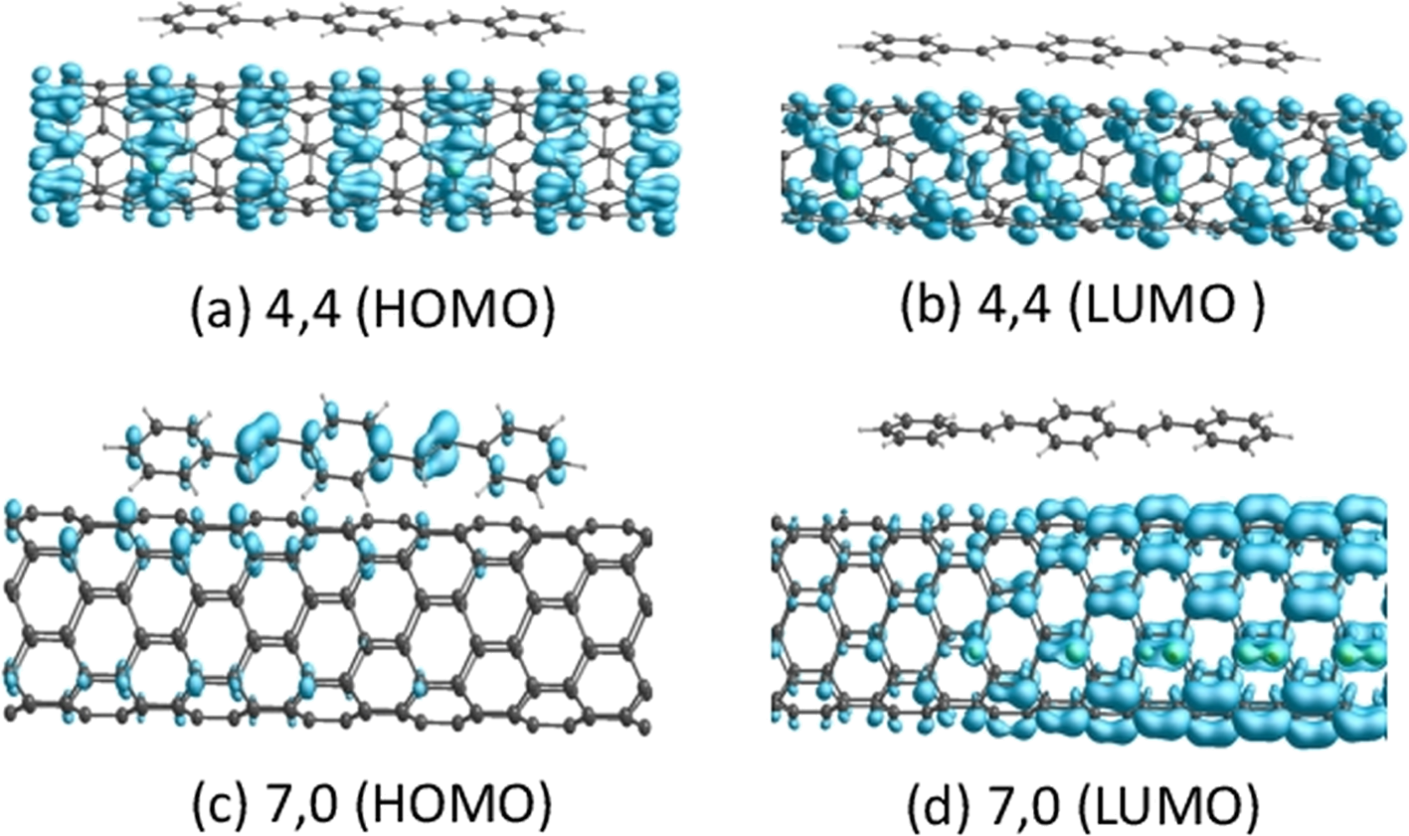
Wavefunction isosurface plots of HOMO and LUMO states for (a,b) (4,4) and (c,d) (7,0) carbon nanotubes interacting with triphenyl PPV oligomer.

The main results of our DFT model are summarized in Figure 2. We indeed find strong interaction between the PPV and both nanotubes with binding energies of 1.21 eV and 0.94 eV for the (4,4) and (7,0) tubes respectively. Figure 2c–f show band structures for the tube segments with and without the PPV. The metallic (4,4) tube is almost unaffected by the interaction. The band structure around the Fermi level remains the same suggesting negligible energy coupling between the two systems in this energy range.However, the semiconducting (7,0) tube shows large coupling between the PPV state near the Fermi level and the NT conduction band state. The system is rendered semi-metallic with a small gap 0.01 eV. However, a total calculated charge transfer of only 0.03*e*, evaluated by Mulliken population analysis, is within the noise level and demonstrates there is negligible charge transfer for either composite at this level of the theory. We note that these are ground state calculations and do not take into account the electron dynamics involved in exciton formation and separation, calculations that are unfortunately inaccessible with our available computing resources. The highest occupied molecular orbital (HOMO) and lowest occupied molecular orbital (LUMO) states for the (7,0) and (4,4) nanotubes in the presence of the triphenyl PPV oligomer are shown in Figure 3. It can be seen that in the presence of the (7,0) tube the system HOMO is primarily localized on the vinyl double bonds along the PPV backbone. Curvature in the PPV oligomer will tend to further localize such states due to decreased coupling between the benzenoid rings and the vinyl states.

The structural rigidity and precise orientation of PPV with respect to the underlying nanotube is likely to vary in realistic experimental samples. Notably, as chain lengths become longer, PPV segments will be less able to position themselves adjacent to, and parallel to, the nanotubes. We explored alternative structural combinations, notably with the triphenyl PPV curved around the nanotubes (radius of curvature 12 Å), and found a metastable minimum only 0.3 eV less stable than the axial configuration described above. This supports the possibility of entropic variations in orientation. While we do not expect this to qualitatively modify the band structures described above, curvature is likely to cause some state localisation and consequent distortion in the PPV bands and hence would be expected to modify primarily the semiconductor SWNT–PPV interactions.

**Figure 4:**
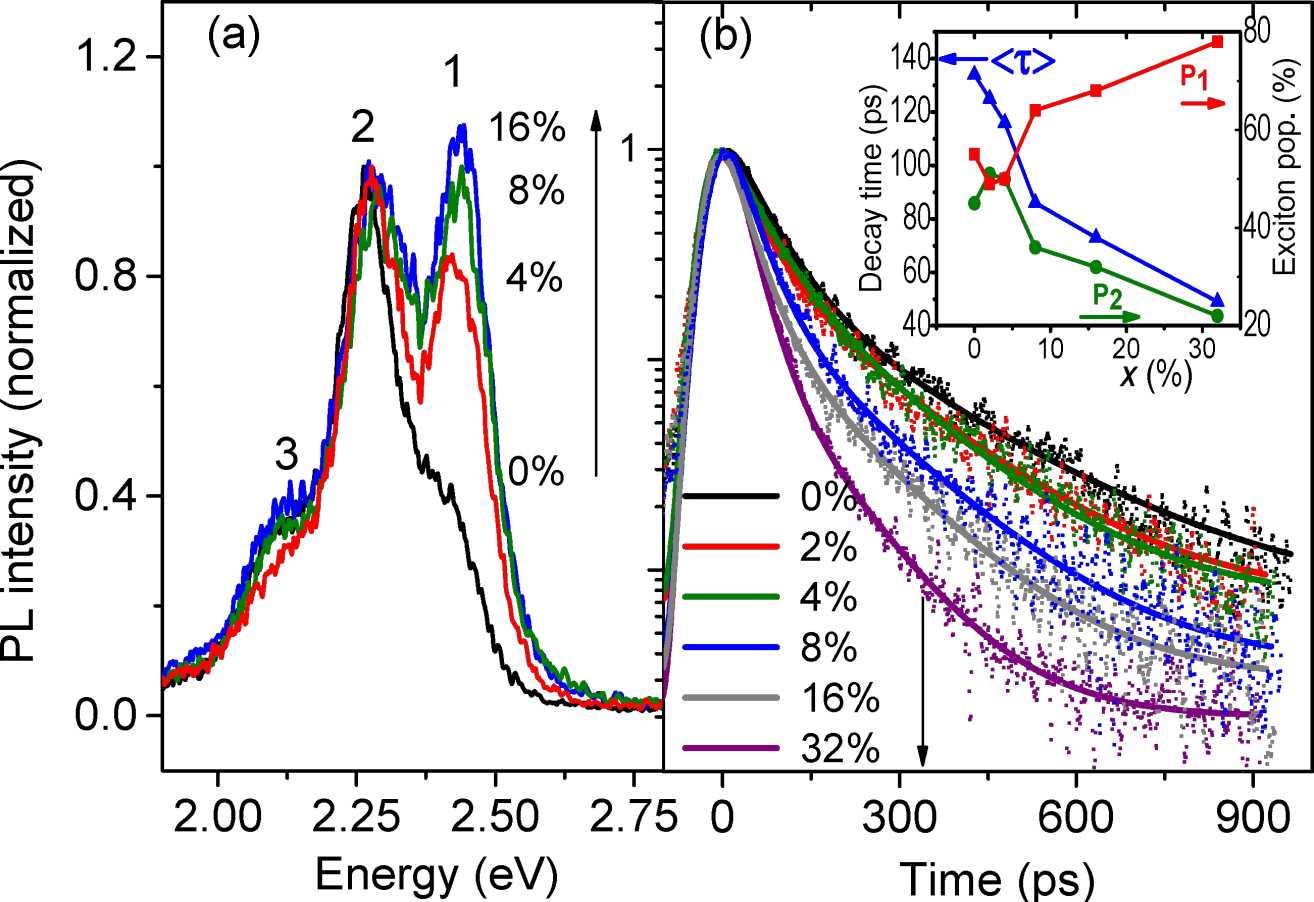
(a) Time-resolved PL spectra of PPV/SWNT composite films for an exciting energy *E*_exc_ = 3.1 eV and for different SWNT mass percentages *x*. (b) Ultrafast PL dynamics at emission 2.25 eV (band 2). Solid lines are fits to the data with the bi-exponential model given by Equation 1 (see text). Inset: average lifetime and relative populations of photoexcitations in the composite films for different mass percentages *x*.

Experimentally, time-resolved PL offers unparalleled possibilities to examine the prediction of a potential energy transfer between the polymer chains and the SWNTs. By resolving the relaxation of photogenerated species this technique can give insight on both radiative and non radiative recombination of charges and their dynamics across polymer–nanotube heterojunctions. The time-resolved PL data taken on the composites are presented in Figure 4a. The high-energy PL band at 2.41 eV, labelled 1, gains intensity with respect to the other lines at 2.26 eV and 2.10 eV (labelled 2 and 3) at high mass percentages *x* of SWNTs in the matrix. As a consequence, although the PL signal becomes weaker as *x* increases, there is a blue shift of the overall remaining emission. A comparable behavior has been observed at the interface of PPV/MWCNT bilayers by Ago et al. [29–30]. This trend is also similar to that of pristine PPV films fabricated from precursor solutions at low concentration or with decreasing conversion temperatures [11]. The inversion of the intensity ratio at *x* = 8% can be explained by the onset of formation of much shorter, conjugated segments with average repeat unit number 3 ≤ *n*_u_ ≤ 5. This intensity ratio also enables rapid determination of the quality of the PPV samples in terms of conjugation and morphology in agreement with previous XRD data [8–10]. Besides, self-absorption is assessed to be a less substantial effect than the salient conjugation changes in the variation of peak-1 intensity with *x*. We note that the persistence of PL for high *x*-values suggests phase segregation between PPV and the SWNT network, likely due to an increase of the SWNT bundle sizes.

To better understand how the morphology influences the excited state dynamics, we have studied the PL intensity decay times of the band 2 (2.25 eV) in the composite films (Figure 4b). The PL kinetics are well reproduced with two exponential decays in a 3-level model comprising the electronic ground state and 2 excited states, according to the following rate equations:

[1]
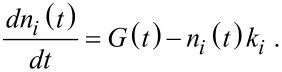


Here, *G*(*t*) is the Gaussian exciton generation function, *n_i_* is the total population of photogenerated charges in the excited energy states *i* = 1 and 2 with initial condition *n_i_*(−∞) = 0, *k_i_* is the inverse of the decay lifetime τ*_i_*. We note that these populations include species recombining radiatively and nonradiatively onto the ground state which is supposed to be negligibly depopulated.

In this elementary model, a fraction of photogenerated charges populating the higher energy level 1 migrate toward the SWNTs (or toward defects for PPV 0%) with a fast thermalization rate 1/τ_1_. This high energy state should be associated with exciton recombination on short segments. The lower energy level 2 typically characterizes long segments (*n_i_* ≥ 5) which are in their most stable state. The charges recombining on them are less mobile due to their low energy and remain on the chains without hopping to the SWNTs. This implies that they survive longer with a slower time constant τ_2_. Therefore, we suggest associating the fast decay component to the PL of PPV, which is quenched by a non-radiative transfer of energy to the polymer network via phonon emission. This process results from the separation of photogenerated excitons at polymer/SWNT interfaces across the heterojunctions. The radiative channel only occurs if the excitons remain trapped on short conjugated segments or migrate on longer PPV segments without encountering the SWNT network. In fact, the migration process is likely to prevent any exciton recombination (in absence of annihilation), therefore raising the probability of exciton separation and promotion of PC.

The total decaying population is 
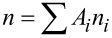
 where the *A_i_* are proportional to the PL intensity from levels *i*. We define an average decay time of the photogenerated charge migration time:

[2]
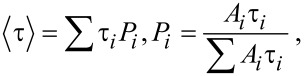


where the weight *P_i_* corresponds to the relative population of photogenerated charges contributing to each of the decay times τ*_i_*.

It is generally accepted that primary photogenerated species in solid-state PPV films under light excitation are in majority intrachain excitons [31]. The simplest approach assumes that the radiative and non-radiative lifetimes τ_r_, τ_nr_ can be expressed as a function of the PL quantum yield *Q*, 
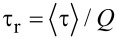
, 
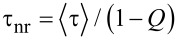
. τ_nr_ accounts for phonon emission, intersystem crossing to triplet states, trapping at chemical defects and exciton migration/dissociation.

For standard PPV (*x* = 0%) we find τ_r_ = 134 ps, τ_nr_ = 705 ps, 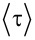 =165 ps very close to the values obtained in the literature [31]. Figure 4 shows that when the percentage of SWNT introduced in the film increases the intensity decay is faster, since 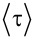 diminishes drastically while the weight *P*_1_ of the first exponential (fast process) concomitantly increases. In addition τ_r_ increases with *x* whereas τ_nr_ decreases, which means that the probability of radiative transitions is lower in heavily loaded composites. The drastic increase of *P*_1_ with *x* shows that the photogenerated species are less localized and bound when the proportion of SWNT in the polymer matrix increases. In the composite the SWNTs operate as exciton dissociation centers. The augmented carrier mobility is confirmed by the photoconductive behavior of the composites and is consistent with our DFT calculations showing strong coupling between PPV and semiconducting SWNT electronic states.

As *x* increases exciton migration under light excitation is favored, resulting in carrier transport on SWNTs and PL quenching. At high SWNT concentrations the PL originates globally from the recombination of excitons on a majority of short conjugated segments, because statistically the proportion of long conjugated segments becomes negligible [11]. These hot excitons can easily migrate due to their high mobility [32], separate when reaching the SWNT network, or transfer energy to SWNTs. This assumption is consistent with the PL quenching, the proportion increase of short conjugated segments [9], the reduction of *Q*, and the rise in photoconductivity when *x* increases, as displayed in Figure 1. Such a picture correlates also very fairly with the prediction of calculation that the short PPV segments couple with each other via the SWNTs.

**Figure 5:**
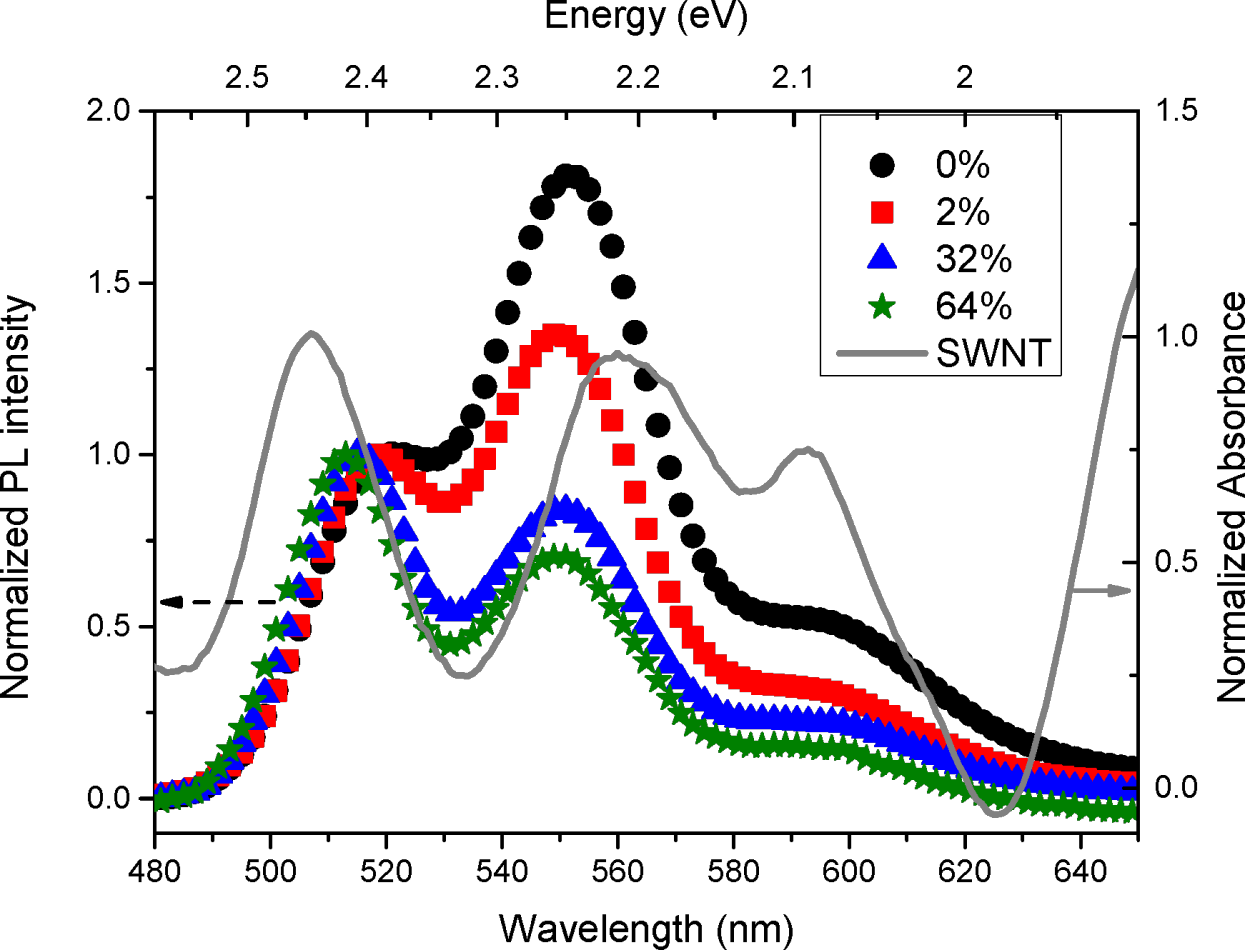
Left side: steady-state PL of PPV/SWNT composite films for different SWNT mass percentages *x*. Right side: absorption spectrum of semiconducting and metallic SWNTs. The excitation energy of emission spectra is *E*_exc_ = 3.1 eV.

Beside hopping of excitations, possible energy transfer between the polymer and the nanotubes is supported by examining steady-state PL energies of a series of composites films containing both metallic and SC nanotubes with *x* increasing from 0% to 64% compared to the optical absorption of SWNTs (Figure 5). We have substracted the absorption background of SWNTs which is due to the π plasmons. In this way, only the contribution of SC and metallic tubes is clearly displayed. These experiments show that there is substantial spectral overlap of PL and optical absorption of SWNTs which could, in principle, favor the occurrence of Förster energy transfer within the separation distance of polymer chains and carbon nanotubes.

## Conclusion

In summary, the PL results suggest sturdy interplay between PPV and semiconducting nanotubes, while photocurrent measurements indicate the key role of metallic nanotubes in the composites at nearly all concentrations above 2%. This is practically confirmed by our DFT calculations predicting strong interaction between PPV and SWNTs, with selective interaction depending on the nature of the nanotube (metallic or semiconducting). Semi-conducting tubes exhibit strong electronic coupling between the nanotube LUMO and the PPV with almost no charge transfer, rendering the tubes semi-metallic and allowing their involvement in dark current charge transfer within the percolative nanotube network. Metallic tubes do not show electronic changes during interaction with PPV. Thus, the calculations suggest that all the nanotubes, both formerly semiconducting (now rendered semi-metallic) and metallic, are implicated in dark current production. Once again, this scenario agrees very well with the photophysical experiments reported here since we observe a rapid quenching of PL signaling energy transfer accompanied by a substantial rise in photoconductivity at increasing fractions of SWNTs in the material.

Finally, the finding that semi-conducting SWNTS play a relevant role in the increase of photoconductivity is supported by their modified electronic band structures in conjunction with the conjugated polymer section. This opens fascinating perspectives to improve exciton dissociation and carrier transport in conjugated polymer composite systems by adapting the polymer properties, for example for solar cell development.
